# Do missing teeth cause early-onset cognitive impairment? Re-examining the evidence using a quasi-natural experiment

**DOI:** 10.1007/s00127-022-02410-y

**Published:** 2022-12-24

**Authors:** Cornelia Santoso, Manuel Serrano-Alarcón, David Stuckler, Stefan Serban, Martin McKee, Attila Nagy

**Affiliations:** 1 Faculty of Public Health, University of Debrecen, Kassai 26, Debrecen, 4012 Hungary; 2 Department of Social and Political Sciences, Bocconi University, Milan, Italy; 3 Department of Dental Public Health, School of Dentistry, University of Leeds, Leeds, UK; 4 Faculty of Public Health and Policy, London School of Hygiene and Tropical Medicine, London, UK

**Keywords:** Tooth loss, Psychiatry, Cognitive impairment, Brain health, Aging

## Abstract

**Purpose:**

Multiple studies have reported a positive association between missing teeth and cognitive impairment. While some authors have postulated causal mechanisms, existing designs preclude assessing this.

**Methods:**

We sought evidence of a causal effect of missing teeth on early-onset cognitive impairment in a natural experiment, using differential exposure to fluoridated water during critical childhood years (ages 5–20 years) in England as the instrument. We coded missing teeth from 0 (≤ 12 missing) to 3 (all missing) and measured the association with cognitive impairment in the English Longitudinal Study of Ageing data (2014–5), covering 4958 persons aged 50–70 years.

**Results:**

We first replicated previous evidence of the strongly positive association of missing teeth with cognitive impairment (*β* = 0.25 [0.11, 0.39]), after adjusting for socio-demographic covariates, such as age, gender, education, and wealth. Using an instrumental variable design, we found that childhood exposure to water fluoridation was strongly associated with fewer missing teeth, with being exposed to fluoridated water during childhood (16 years) associated with a 0.96 reduction in the missing teeth scale (*β* = − 0.06 [− 0.10, − 0.02]). However, when using the instrumented measure of missing teeth, predicted by probability of fluoride exposure, we found that missing teeth no longer had an association with cognitive impairment (*β* = 1.48 [− 1.22, 4.17]), suggesting that previous oral health-cognitive impairment associations had unobserved confounding.

**Conclusions:**

Our findings are consistent with the possibility that unobserved confounding leads to the oft-observed association between missing teeth and early-onset cognitive impairment, suggesting that the relationship is spurious rather than causal.

**Supplementary Information:**

The online version contains supplementary material available at 10.1007/s00127-022-02410-y.

## Introduction

Dementia and cognitive impairment are among the leading pressing healthcare challenges as populations age [1], with dementia affecting over 55 million people in 2019, or 0.7% of the world’s population, and it is projected to rise to over 130 million in 2050 [2–4]. Its cost, worldwide, was estimated at US$ 1.3 trillion in 2019, increasing to US$ 2.8 trillion USD in 2030 [2]. It is, therefore, of utmost importance to identify ways to prevent them.

Tooth loss has been suggested as one potential risk factor for early-onset cognitive impairment and dementia [5]. Numerous studies have reported associations between tooth loss and neurocognitive disorders [5, 6]. It has been argued that these associations are biologically plausible. Several mechanisms have been proposed. One involves masticatory dysfunction, contributing to nutritional deficiencies, which may in turn affect central nervous system (CNS) or brain health [5, 7]. Another is where tooth loss reduces interocclusal contacts (contacts between teeth in the upper and lower jaws), providing less somatosensory feedback to the CNS, compromising cognition in a manner similar to how sensory impairment from loss of vision and hearing impairs cognition [7]. A third invokes the periodontal inflammation that precedes tooth loss, which may affect the CNS and compromise cognition [5, 7].

On the other hand, some longitudinal studies and systematic reviews have reported mixed findings [8, 9]. For example, Tsakos et al. (2015) found, in a longitudinal study conducted in England, that more missing teeth predicted greater risk of worsening cognitive function, but in people aged 60–74 and not in those 75+ [10]. Several prospective studies in Japan did not find any association with cognitive impairment [11] or dementia [12] and neither did a study among women in Sweden [13] or in California [14].

At present, therefore, we cannot say whether the association between missing teeth and cognitive impairment is causal. This will require new research that addresses the two major limitations of the existing studies. These are the risk of unmeasured confounders and the potential for reverse causality, whereby people with cognitive impairment might have more risk factors for poor oral health, leading to a higher number of missing teeth [5, 15]. Thomson and Barak (2021) argued, for example, that none of the proposed biological mechanisms is currently supported by convincing empirical evidence. They contend that earlier evidence linking oral health and cognitive outcomes is likely an artefact of residual confounding from factors such as smoking, nutrition, lifestyle, or other common risk factors acting throughout the life course. They also expressed concern that claims of causality might be abused by dentists seeking to promote prosthodontic interventions in vulnerable older persons [7].

Here, we employ the approach of a study that seeks to address the potential for unmeasured confounders and reverse causality. The study took advantage of a natural experiment whereby different parts of the UK introduced water fluoridation at different points between 1964 and 1987 [16–18]. This design enables us to determine whether missing teeth are likely to be causally linked to early-onset cognitive impairment.

## Methods

Following best practice, we adhered to the Strengthening the Reporting of Observational Studies in Epidemiology (STROBE) guidelines for cross-sectional studies [19].

### Data source and study population

Our data were taken from the English Longitudinal Study of Ageing (ELSA), a prospective cohort study of non-institutionalised persons aged 50 + and their partners in England. The survey received ethical approval from the National Health Service (NHS) Research Ethics Committees under the National Research and Ethics Service (NRES). Details of the survey have been described elsewhere [20]. Since this study is a secondary analysis of publicly available data, a separate ethical approval is not needed.

**Fig. 1:**
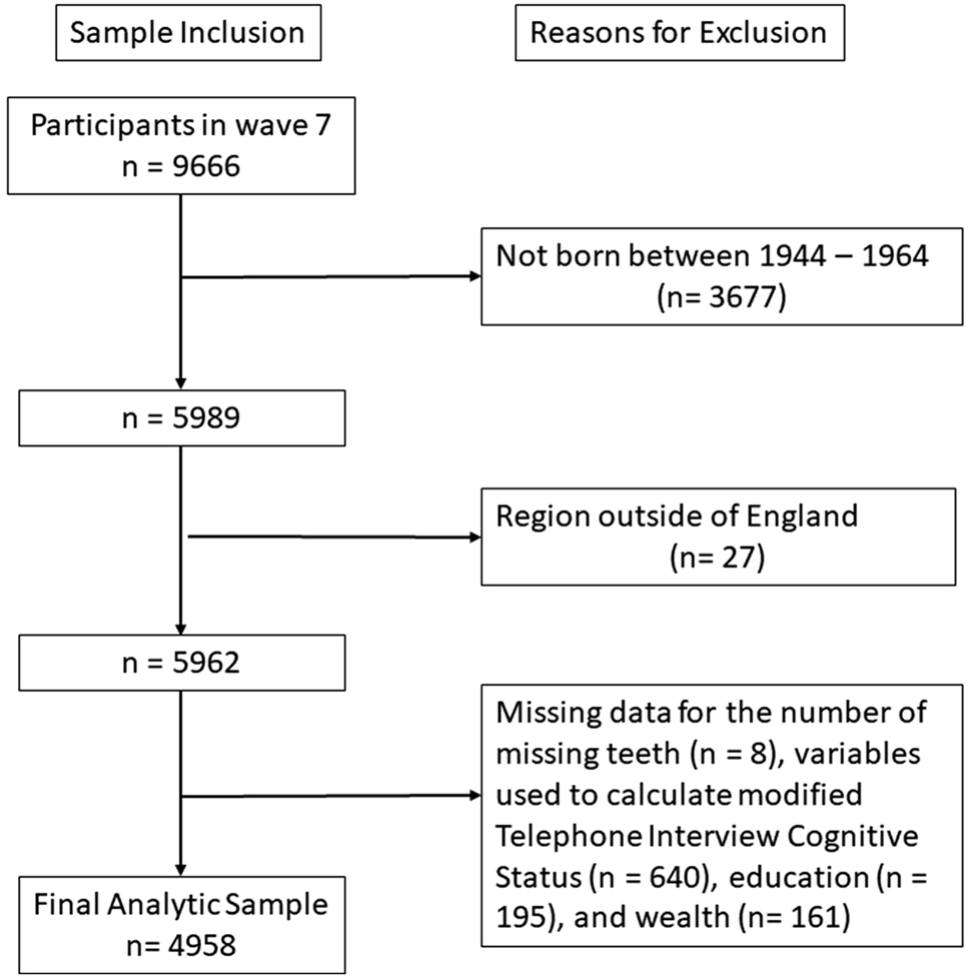
Flowchart of the study participants

Briefly, we used wave 7 cross-sectional data, which were collected between June 2014 and May 2015 [20]. Figure 1 shows the flowchart of the study participants and sample inclusion. Of the 9666 participants, 5989 were born between 1944 and 1964. After excluding those whose region was outside of England where fluoridation took place (*n* = 27) and those with missing data on variables used in this study (*n* = 1004), we had a final analytical sample of 4958 adults.

### Cognitive performance measurements

We captured cognitive function using an established index of cognitive performance, the modified Telephone Interview for Cognitive Status (mTICS). This is a 27-point scale that sums the following test scores: immediate 10 word-list recall (10 points), delayed 10-world list recall (10 points), counting backwards (2 points), and serial 7 subtraction (5 points) [21, 22]. With immediate and delayed 10 word-list recall and serial 7 subtraction, one point is given for each correct answer. With counting backwards, two points were given if an individual could successfully do so at the first attempt, while one point was given for success at the second attempt [20]. Thus, in the original scale, higher scores represent better cognitive function. However, to facilitate easier interpretation, we reverse-coded the original scale so that higher scores correspond to greater cognitive impairment (0 best—27 worst). For our primary analyses, we treated this as a continuous variable. In a sensitivity analysis we also dichotomised this variable as follows: normal cognition (coded as 0) and cognitive impairment or probable dementia (coded as 1). Following a threshold employed by prior studies [21, 22], normal cognition was defined if the original mTICS score was 12–27, and cognitive impairment or probable dementia if the original score was 0–11 [21, 22].

### Self-Reported missing teeth measurement

ELSA survey respondents were asked to indicate their number of remaining teeth, with the four following response options: no teeth, 1–9 teeth, 10–19 teeth, or 20 teeth or more [20]. In our study, this variable was coded from 0 to 3, corresponding to 0 =  ≤ 12 missing teeth, 1 = 13–22 missing teeth, 2 = 23–31 missing teeth, and 3 = 32 missing teeth. Therefore, a higher score corresponded to a higher number of missing teeth.

### Covariate measurements

Replicating prior studies, we adjusted our models for socio-demographic covariates, including age, gender, education, total net wealth, and region of residence. Age was calculated by subtracting birth year of the participants from the interview year of 2014. Educational level was defined by the highest educational qualification, categorised into the following three groups: low (‘no qualification’), medium (‘NVQ1/CSE other grade equivalent qualification’, ‘NVQ2/GCE O-level equivalent qualification’, ‘NVQ3/GCE A-level equivalent qualification’, ‘foreign/other’), and high (‘higher education below degree’, ‘NVQ4/NVQ5/Degree or equivalent qualification’). The derived variable of total net wealth at benefit unit level (referring to “a couple or a single person with any dependent children they may have”) was provided by the survey, which was the sum of savings, investments, physical wealth, and housing wealth after subtracting financial debt and mortgage debt [20]. We used this derived variable to divide the sample into five wealth quantiles. Region of residence in wave 7 was considered as proxy for the region where the participants had lived in childhood. In this respect, we followed a previous study using the data that found a strong association between loss of teeth, estimated by the probability of fluoridation of area of residence, and ability to perform activities of daily life in England [16]. The authors of that study considered the role of mobility but set out arguments as to why any effect was likely to be non-differential.

### Natural experiment design

To help causality, this study employed a natural experiment design used in a prior study of oral health [16]. Briefly, we exploit the fact that community water fluoridation schemes were implemented across administrative regions of England in a staggered manner. Individuals in our sample will have been differently exposed to fluoridated water depending on their year and region of birth. We then construct the instrument variable (IV) as the total annual probability of childhood exposure to naturally and/or artificially fluoridated tap water between the age of 5 and 20 years. This was the period from eruption until post-eruptive enamel maturation of permanent teeth, which was considered to be susceptible to the preventive action of fluoride. The annual probability of being exposed to fluoride was calculated in each region by dividing the annual number of people covered by water fluoridation with the population size of the region in 2012 [16]. Data on the number of people covered by fluoridated water and the starting year of artificial water fluoridation was taken from the earlier studies that used these data [17, 18]. If a range of years was given, we took the midpoint as the initiation year of the fluoridation scheme.

**Fig. 2:**
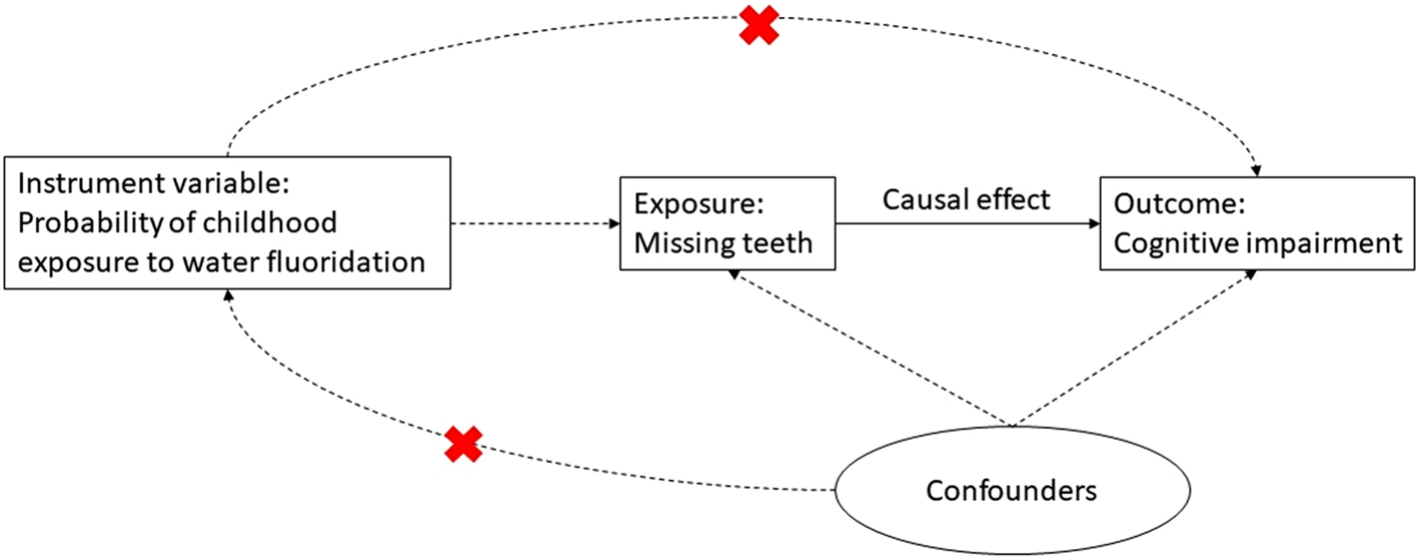
Conceptual framework of the instrumental variable analysis. Briefly, the instrument variable should be independent from the confounders, and should only affect the outcome through the pathway of the exposure variable [16]. The concept of the two-stage least square regression is that the exposure (missing teeth) is first regressed on the instrument variable (probability of childhood exposure to water fluoridation). The resulting fitted value of missing teeth is then used to estimate the causal effect between missing teeth and cognitive impairment

Figure 2 shows the conceptual framework of instrumental variable analysis. In brief, instrumental variable analysis is a method to control for unobserved confounders in observational studies, which is increasingly used in epidemiology and health service research [23]. Similar to a prior study of oral health [16], our instrumental variable design should comply with the following two conditions:

1. The ‘strong first stage’: water fluoride (instrument variable) should strongly affect tooth loss. The protective effect of fluoride in childhood on risk of dental caries is very well-documented and established in the scientific literature [24, 25]. We test for this in our model.
2. The ‘exclusion restriction’: childhood exposure to water fluoridation influenced cognitive impairment in adulthood only through the pathway of tooth loss, and not through any other pathway. This cannot be tested empirically but it is an assumption supported by the literature. Although there has been speculation about potential cognitive effects of fluoride, at present there is no or insufficient evidence that fluoride has adverse impacts on cognitive impairment in adulthood, cognitive development or intelligence quotient (IQ) [26–29]. Current acceptable concentration levels of fluoride in drinking water (i.e., natural water fluoridation 0.5–1.5 ppm, targeted artificial water fluoridation 1.0 ppm) were the levels employed in this study [16, 27].

Additionally, the instrument variable should be as good as randomly assigned, conditional on our control variables. That is, it should not be associated with any individual characteristics that might affect cognitive impairment. In Supplementary Table S1, we test for this, and we show that exposure to fluoride (instrument variable) is associated only with the region and age, which indeed makes sense since the rollout of fluoride was staggered by region. To control for this, we adjust for region and age in our preferred model. We also limited our sample to persons aged 50–70 years (roughly equivalent to those born between 1944 and 1964). Persons born in 1944 were the first cohort whose childhood was affected by the first fluoridation scheme in England, which was started in 1964 [17, 18].

### Statistical analyses

We operationalised the instrumental variable model, as follows:

1a. ${\mathrm{Missing teeth}}_{\mathrm{i}}$= ${\alpha }_{0}$ + ${\alpha }_{1}$ Fluoride + *γ *SocDem + ${\alpha }_{2}$ Region + ${\varepsilon }_{i}$ (First stage).

1b. ${\mathrm{Early cognitive impairment}}_{i}$ = ${\beta }_{0}$ + ${\beta }_{1}$ Predicted Missing Teeth + *µ *SocDem + ${\beta }_{2}$ Region + ${u}_{i}$ (Second stage).

On the first stage regression, we estimate the number of missing teeth ($$Missing teeth)$$ with respect to our instrumental variable (Fluoride, i.e. the total annual probability of fluoride exposure).

Predicted missing teeth concept is derived from the predicted value of model 1a and included in model 1b. ${\beta }_{1}$ measures the causal effect of missing teeth on early cognitive impairment. We further control for a vector of socio-demographical control variables, including age, gender, education, and wealth (SocDem) and Region dummies at NUTS3 level (*Region*). Indeed, a “balance test” found substantial differences among those with missing and without missing teeth, suggesting the importance of controlling for these variables in the model, and the use of the instrumental variable (Supplementary Table S2). Standard errors (SE) were clustered by age and region to reflect non-independence of sampling. All analyses were conducted unweighted using STATA 17.0 [30].

## Results

### Descriptive statistics

Table 1 shows the characteristics of the study participants. The mean (± SD) of age of the sample was 62.0 (± 5.2). Most of them were females, had intermediate education, resided in South East region, and had ≤ 12 missing teeth. The mean (± SD) of cognitive impairment score was 8.8 (± 3.5) and around 4% was considered having cognitive impairment or probable dementia. According to bivariate analyses, those with older age, male gender, low educational level and economic status, or a higher number of missing teeth tended to have a higher mean cognitive impairment score.

**Table 1:** Characteristics of the study participants

Variables	*N* (%) or Mean (± SD)	Mean (± SD) cognitive impairment score	*P*-value
Age group			
50–60	1872 (37.8%)	8.7 (± 3.7)	0.008 ^†^
61–70	3086 (62.2%)	8.9 (± 3.4)	
Gender			
Female	2762 (55.7%)	8.5 (± 3.6)	< 0.001 ^†^
Male	2196 (44.3%)	9.2 (± 3.4)	
Education			
No qualification	773 (15.6%)	10.8 (± 3.7)	< 0.001 ^ǂ^
Intermediate	2494 (50.3%)	8.9 (± 3.3)	
High	1691 (34.1%)	8.0 (± 3.3)	
Wealth			
Lowest quantile	993 (20.0%)	10.3 (± 3.7)	< 0.001 ^ǂ^
2nd quantile	992 (20.0%)	9.4 (± 3.4)	
3rd quantile	991 (20.0%)	8.7 (± 3.4)	
4th quantile	991 (20.0%)	8.2 (± 3.2)	
Highest quantile	991 (20.0%)	7.7 (± 3.3)	
Region			
North East	289 (5.8%)	9.0 (± 3.4)	0.050 ^ǂ^
North West	622 (12.6%)	8.8 (± 3.7)	
Yorkshire and The Humber	498 (10.0%)	9.0 (± 3.5)	
East Midlands	558 (11.3%)	8.8 (± 3.5)	
West Midlands	547 (11.0%)	9.0 (± 3.4)	
East of England	616 (12.4%)	8.9 (± 3.4)	
London	445 (9.0%)	9.3 (± 4.0)	
South East	829 (16.7%)	8.6 (± 3.5)	
South West	554 (11.2%)	8.6 (± 3.3)	
Cognitive impairment or probable dementia			
Yes	200 (4.0%)	17.6 (± 2.3)	< 0.001 ^†^
No	4758 (96.0%)	8.5 (± 3.1)	
Cognitive impairment score	8.8 (± 3.5)	N/A	N/A
Number of missing teeth (categories)			
≤ 12	3820 (77.1%)	8.5 (± 3.4)	< 0.001 ^ǂ^
13–22	657 (13.3%)	9.7 (± 3.6)	
23–31	261 (5.3%)	10.4 (± 3.7)	
32	220 (4.4%)	10.1 (± 3.6)	
Total annual likelihood of childhood fluoride exposure	0.47 (± 1.0)	−0.005 ^§^	0.716 ^§^

Cognitive impairment scores were derived from the reverse-coded modified Telephone Interview Cognitive Score (continuous), from 0 (best) to 27 (worst)

*N/A* Not applicable

^†^*P* value was based on Mann–Whitney test

^ǂ^*P* value was based on Kruskall–Wallis test

^§^Pearson correlation coefficient was used to measure the strength and significance of correlation between two continuous variables

### Replicating prior studies

First, we sought to reproduce prior studies reporting a positive association of missing teeth with early-onset cognitive impairment. Table 2 shows the results of these Ordinary Least Squares (OLS) regressions. As shown, we observed that the number of missing teeth was strongly and positively associated with a higher score of cognitive impairment (*p* value < 0.001). Similar results can also be seen when using a binary measure of cognitive impairment and probable dementia (Supplementary Table S3).

**Table 2:** Ordinary Least Squares estimation with cognitive impairment score (continuous) as the outcome

	Model 1	Model 2	Model 3	Model 4
	*β* (SE)	*β* (SE)	*β* (SE)	*β* (SE)
Number of missing teeth	0.71 (0.07)***	0.70 (0.07)***	0.25 (0.07)***	0.25 (0.07)***
Region dummies	Adjusted	Adjusted	Adjusted	Adjusted
Gender				
Female	Ref	Ref	Ref	Ref
Male	0.67 (0.09)***	0.68 (0.08)***	0.79 (0.09)***	0.81 (0.09)***
Education				
No qualification			Ref	Ref
Intermediate			− 1.43 (0.16)***	− 1.43 (0.16)***
High			− 2.05 (0.17)***	− 2.02 (0.17)***
Wealth				
Lowest quantile			Ref	Ref
2nd quantile			− 0.56 (0.16)***	− 0.55 (0.15)***
3rd quantile			− 1.20 (0.16)***	− 1.17 (0.16)***
4th quantile			− 1.65 (0.15)***	− 1.59 (0.16)***
Highest quantile			− 2.12 (0.18)***	− 2.08 (0.18)***
Age (continuous)	0.02 (0.01)*		0.04 (0.01)***	
Age dummies		Adjusted		Adjusted
N	5314	5314	4958	4958

Cognitive impairment scores were derived from the reverse-coded modified Telephone Interview Cognitive Score (continuous), from 0 (best) to 27 (worst). Clustered standard errors by age and region in parentheses. *p < 0.05, **p < 0.01, ***p < 0.001

### Instrumental variable design

Next, we turned to implementing the causal design using instrumental variable modelling. Table 3 shows the first-stage results of instrumental variable regression, testing validity of the fluoride instrumental design. As anticipated, it showed that the greater probability of exposure to water fluoridation in childhood years were strongly associated with fewer missing teeth (*p* value < 0.01). This further supported our first assumption, that childhood exposure to water fluoridation was a protective factor for tooth loss in adulthood.

**Table 3:** Two-Stage Least Squares estimation of first stage regression with missing teeth as the outcome

	Model 1	Model 2	Model 3	Model 4
	*β* (SE)	*β* (SE)	*β* (SE)	*β* (SE)
Total annual likelihood of childhood fluoride exposure	− 0.06 (0.02)**	− 0.07 (0.02)***	− 0.06 (0.02)**	− 0.06 (0.02)**
Region dummies	Adjusted	Adjusted	Adjusted	Adjusted
Gender				
Female	Ref	Ref	Ref	Ref
Male	− 0.00 (0.02)	− 0.00 (0.02)	0.02 (0.02)	0.02 (0.02)
Education				
No qualification			Ref	Ref
Intermediate			− 0.24 (0.04)***	− 0.24 (0.04)***
High			− 0.35 (0.04)***	− 0.36 (0.04)***
Wealth				
Lowest quantile			Ref	Ref
2nd quantile			− 0.34 (0.04)***	− 0.35 (0.04)***
3rd quantile			− 0.43 (0.04)***	− 0.43 (0.04)***
4th quantile			− 0.53 (0.04)***	− 0.53 (0.04)***
Highest quantile			− 0.56 (0.04)***	− 0.56 (0.04)***
Age (continuous)	0.02 (0.00)***		0.03 (0.00)***	
Age dummies		Adjusted		Adjusted
*N*	5314	5314	4958	4958
*F*-statistic	10.28	12.64	9.54	11.13

Clustered standard errors by age and region in parentheses. *p < 0.05, **p < 0.01, ***p < 0.001

We then operationalised the missing teeth which were attributable to lower-level probability of fluoride exposure in the main linear model for early onset cognitive impairment. Table 4 shows the results of this second stage of the instrumental variables regression. Once using fluoride as an instrument variable, there was no longer an association between missing teeth and cognitive impairment (*p* = 0.28). Similar results can also be seen when using a binary measure of cognitive impairment and probable dementia (Supplementary Table S4).

**Table 4:** Two-Stage Least Squares estimation of second stage regression with cognitive impairment score (continuous) as the outcome

	Model 1	Model 2	Model 3	Model 4
	*β* (SE)	*β* (SE)	*β* (SE)	*β* (SE)
Number of missing teeth	0.97 (1.60)	1.93 (1.32)	0.42 (1.65)	1.48 (1.38)
Region dummies	Adjusted	Adjusted	Adjusted	Adjusted
Gender				
Female	Ref	Ref	Ref	Ref
Male	0.68 (0.08)***	0.68 (0.08)***	0.79 (0.09)***	0.79 (0.09)***
Education				
No qualification			Ref	Ref
Intermediate			− 1.39 (0.43)**	− 1.13 (0.37)**
High			− 1.99 (0.60)***	− 1.58 (0.51)**
Wealth				
Lowest quantile			Ref	Ref
2nd quantile			− 0.50 (0.61)	− 0.13 (0.51)
3rd quantile			− 1.12 (0.75)	− 0.64 (0.64)
4th quantile			− 1.56 (0.89)	− 0.94 (0.74)
Highest quantile			− 2.02 (0.96)*	− 1.40 (0.80)
Age (continuous)	0.02 (0.04)		0.04 (0.05)	
Age dummies		Adjusted		Adjusted
*N*	5314	5314	4958	4958
*F*-statistic	10.28	12.64	9.54	11.13

Cognitive impairment scores were derived from the reverse-coded modified Telephone Interview Cognitive Score (continuous), from 0 (best) to 27 (worst). Clustered standard errors by age and region in parentheses. *p < 0.05, **p < 0.01, ***p < 0.001

These patterns were consistent with (Models 3 and 4) or without adjustment (Models 1 and 2) for socio-demographic confounders.

## Discussion

Our study investigated the *causal effect* of missing teeth on early-onset cognitive impairment. We employed a natural-experiment design with data from areas of England that benefited from community water fluoridation schemes which began in 1964. Our study was able to replicate previous studies which observed that the number of missing teeth was significantly and positively associated with cognitive impairment. However, when using an instrumental variable technique based on water fluoridation to adjust for potential unobserved confounding factors, we found that missing teeth no longer had an association with cognitive impairment.

Before interpreting our findings further, we must note several important methodological limitations arising from the survey and the natural experiment design. First, the number of missing teeth was self-reported, which might lead to inaccuracy and measurement errors. Additionally, the numbers were categorised rather than coded as a continuous variable, which further could lead to non-differential measurement error. Second, we limited the sample to those aged 50–70 and their partners, reflecting early-onset cognitive impairment. This study may not, therefore, applicable to dementia, and future research would be needed to investigate this issue in older populations. Third, our analysis was conducted using unweighted data and, while this does not affect the validity of our estimates, it makes the sample no longer representative of older adults in England as a whole.^1^

A considerable strength of our study was employing a natural experiment, based on community water fluoridation schemes, which is well established as a critical intervention to improve population oral health [16]. The use of instrumental variable methods in this field is novel and can address methodological limitations in existing oral-cognitive health research, particularly unobserved confounders and reverse causality. For example, very few existing studies on this topic have included nutrition as a potential confounding factor [8, 9].

Notwithstanding the strengths of this design over conventional approaches, it also has limitations which are important to mention. First, we used an ecological measure of fluoride exposure, which was assessed only by tap water fluoridation. There are, however, other sources of fluoride to which individuals can be exposed, such as the use of fluoridated toothpaste or other dental products, consumption of food and beverages high in fluoride (e.g., tea and marine fishes), and high natural fluoride levels in some areas [16, 27]. Second, using current residency as a proxy for childhood residency might lead to non-differential misclassification bias, so affecting the estimated link between fluoride and missing teeth. This argument was also supported by the previous study employing the same study design in England [16]. Third, data on fluoride exposure were at the county level, which we aggregated to the regional level. However, in reality the probability of fluoride exposure in individuals was not likely to be equally distributed within a region. If this led to substantial dilution, however, we would not be able to identify a strong association between fluoridation and fewer missing teeth. Our observation of a strong association, as anticipated, between fluoride and missing teeth, helps establish the validity of the methodological approach for testing our hypothesis. Nonetheless, future research would ideally capture exposure to fluoridation at more disaggregated, local levels. Fourth, tooth loss can be an indicator of exposure of both past dental caries and/or past periodontal disease. Thus, the approach of using community water fluoridation as an instrument variable for missing teeth captures lifetime dental caries experience instead of periodontal diseases [16]. It is possible that periodontal inflammation which might initiate systemic inflammatory processes might still be associated with cognitive function in the long-term. However, our study was able to demonstrate that missing teeth per se were not a causal factor.

Notwithstanding these limitations, our study is among the few to use a natural experiment, testing the causal evidence of the effect of the number of missing teeth on cognitive impairment. The instrumental variable approach has several advantages over other panel data approaches that previous studies on this topic have employed [10, 15].

Our findings are consistent with the hypothesis that the observed association of tooth loss and cognitive impairment found in prior studies might be due to unmeasured confounders and reverse causality. These corroborate the views of Thomson and Barak [7], who took a life course perspective, arguing that tooth loss shares common risk factors with cognitive impairment, such as low socioeconomic status (SES) and education, personality characteristics, smoking, and diabetes. They further argued that it is childhood cognitive ability that serves as an initial antecedent for a spurious missing teeth-cognitive impairment association. People with higher cognitive function in childhood have greater opportunities to have better oral health through the life course. They also tend to have greater cognitive reserve, and thus function, in later life. For example, they are more likely to have more resources to access dental care, as well as better ability to make healthier lifestyle choices (e.g., healthier diet, less smoking, better self-care) that reduce the risk of both tooth loss and cognitive impairment on their life course journey [7]. Therefore, public health interventions addressing psychosocial and lifestyle factors across the life course might not only protect against dementia but also bring other health benefits, including cardiovascular health and general well-being [31], among others.

Our study points to several directions for future research. While we have demonstrated that missing teeth per se are not associated with cognitive impairment, we cannot rule out that periodontal inflammation might affect cognition. Future research, ideally drawing on available longitudinal studies, could further evaluate this hypothesis. Importantly, this would need to take into account the potential risks of bias facing studies of oral health, including adjusting for smoking [7] and unhealthy diet, and the potential for reverse causality. One approach which holds great potential would be to re-evaluate existing randomised controlled trials (RCTs) of periodontal therapy to examine whether they had unintended knock-on causal improvements in cognitive health [32].

In summary, our study does not find evidence to support a causal impact of missing teeth on early-onset cognitive impairment. Rather, our findings are consistent with the hypothesis that social and health-related factors across the life-course are common to tooth loss and cognitive impairment. There may be untapped potential for dentists to work together with other health professionals to act on earlier life-course determinants to prevent cognitive impairment and promote healthier ageing.

### Supplementary Information

Below is the link to the electronic supplementary material.

Supplementary file1 (DOCX 25 KB)

## Data Availability

The English Longitudinal Study of Ageing (ELSA) data can be accessed through UK Data Service portal. Procedure to access the data can be found at: https://www.elsa-project.ac.uk/accessing-elsa-data.

## Footnotes

1 The cross-sectional weight provided by the survey was only available for core members [20].
