# Supplementary material for: Do missing teeth cause early-onset cognitive impairment? Re-examining the evidence using a quasi-natural experiment
Source: Soc Psychiatry Psychiatr Epidemiol. 2022 Dec 24;59(4):705–14. doi: 10.1007/s00127-022-02410-y (PMC10960740; doi:10.1007/s00127-022-02410-y)
Supplement: Supplementary file 1 — Supplementary file1 (DOCX 25 KB) [file 127_2022_2410_MOESM1_ESM.docx]

Supplementary Table S1. Random assignment test with total annual probability of fluoride as the outcome

|  | **Model 1** | **Model 2** |
| --- | --- | --- |
|  | **ß (SE)** | **ß (SE)** |
| Gender |  |  |
| Female | Ref | Ref |
| Male | -0.00 (0.01) | 0.00 (0.01) |
| Education |  |  |
| No qualification | Ref | Ref |
| Intermediate | 0.02 (0.02) | 0.02 (0.02) |
| High | 0.02 (0.02) | 0.02 (0.02) |
| Wealth |  |  |
| Lowest quantile | Ref | Ref |
| 2nd quantile | -0.06 (0.03)* | -0.06 (0.03)* |
| 3rd quantile | -0.03 (0.03) | -0.03 (0.03) |
| 4th quantile | -0.00 (0.03) | 0.01 (0.03) |
| Highest quantile | -0.00 (0.02) | 0.01 (0.02) |
| Region dummies |  |  |
| North East | Ref | Ref |
| North West | -2.52 (0.28)*** | -2.51 (0.28)*** |
| Yorkshire and The Humber | -2.55 (0.28)*** | -2.54 (0.28)*** |
| East Midlands | -2.52 (0.28)*** | -2.51 (0.28)*** |
| West Midlands | -0.71 (0.34)* | -0.71 (0.33)* |
| East of England | -2.57 (0.28)*** | -2.56 (0.28)*** |
| London | -2.37 (0.28)*** | -2.36 (0.28)*** |
| South East | -2.69 (0.28)*** | -2.69 (0.28)*** |
| South West | -2.66 (0.28)*** | -2.66 (0.28)*** |
| Age (continuous) | -0.06 (0.01)*** |  |
| Age dummies |  |  |
| 50 |  | Ref |
| 51 |  | -0.24 (0.59) |
| 52 |  | -0.38 (0.55) |
| 53 |  | -0.41 (0.57) |
| 54 |  | -0.50 (0.54) |
| 55 |  | -0.55 (0.53) |
| 56 |  | -0.66 (0.51) |
| 57 |  | -0.70 (0.50) |
| 58 |  | -0.82 (0.48) |
| 59 |  | -0.95 (0.47)* |
| 60 |  | -1.01 (0.47)* |
| 61 |  | -1.06 (0.47)* |
| 62 |  | -1.11 (0.47)* |
| 63 |  | -1.15 (0.47)* |
| 64 |  | -1.21 (0.48)* |
| 65 |  | -1.24 (0.48)* |
| 66 |  | -1.28 (0.48)** |
| 67 |  | -1.29 (0.49)** |
| 68 |  | -1.37 (0.50)** |
| 69 |  | -1.40 (0.51)** |
| 70 |  | -1.43 (0.52)** |
| N | 4958 | 4958 |

Note: Clustered standard errors by age and region in parentheses. * p<0.05, ** p<0.01, *** p<0.001

Supplementary Table S2. Balance test of the number of missing teeth by characteristics of the participants

|  | **Treated group (≤12 teeth)** | **Control group (≥13 teeth)** | **P-value** |
| --- | --- | --- | --- |
|  | **N (%) or mean (±SD)** | **N (%) or mean (±SD)** |  |
|  | **N = 3820 (77.0%)** | **N = 1138 (23.0%)** |  |
| Age | 61.6 (±5.3) | 63.5 (±4.8) | <0.001^ǂ^ |
| Gender |  |  |  |
| Female | 2152 (77.9%) | 610 (22.1%) | 0.103^†^ |
| Male | 1668 (76.0%) | 528 (24.0%) |  |
| Education |  |  |  |
| No qualification | 444 (57.4%) | 329 (42.6%) | <0.001^†^ |
| Intermediate | 1904 (76.3%) | 590 (23.7%) |  |
| High | 1472 (87.1%) | 219 (13.0%) |  |
| Wealth |  |  |  |
| Lowest quintile | 559 (56.3%) | 434 (43.7%) | <0.001^†^ |
| 2^nd^ quintile | 731 (73.7%) | 261 (26.3%) |  |
| 3^rd^ quintile | 800 (80.7%) | 191 (19.3%) |  |
| 4^th^ quintile | 851 (85.9%) | 140 (14.1%) |  |
| Highest quintile | 879 (88.7%) | 112 (11.3%) |  |
| Region |  |  |  |
| North East | 197 (68.2%) | 92 (31.8%) | <0.001^†^ |
| North West | 449 (72.2%) | 173 (27.8%) |  |
| Yorkshire and The Humber | 363 (72.9%) | 135 (27.1%) |  |
| East Midlands | 421 (75.5%) | 137 (24.6%) |  |
| West Midlands | 421 (77.0%) | 126 (23.0%) |  |
| East of England | 486 (78.9%) | 130 (21.1%) |  |
| London | 360 (80.9%) | 85 (19.1%) |  |
| South East | 659 (79.5%) | 170 (20.5%) |  |
| South West | 464 (83.8%) | 90 (16.3%) |  |
| Cognitive impairment score | 8.5 (±3.4) | 9.9 (±3.7) | <0.001^ǂ^ |
| Cognitive impairment and probable dementia |  |  |  |
| Yes | 110 (55.0%) | 90 (45.0%) | <0.001^†^ |
| No | 3710 (78.0%) | 1048 (22.0%) |  |

Note: Cognitive impairment scores were derived from the reverse-coded modified Telephone Interview Cognitive Score (continuous), from 0 (best) to 27 (worst).

^†^ P-value was based on chi-square test

^ǂ^ P-value was based on Mann-Whitney test.

Supplementary Table S3. Ordinary Least Squares estimation with cognitive impairment and probable dementia (binary) as the outcome

|  | **Model 1** | **Model 2** | **Model 3** | **Model 4** |
| --- | --- | --- | --- | --- |
|  | **ß (SE)** | **ß (SE)** | **ß (SE)** | **ß (SE)** |
| Number of missing teeth | 0.03 (0.01)*** | 0.03 (0.01)*** | 0.02 (0.01)** | 0.02 (0.01)** |
| Region dummies | Adjusted | Adjusted | Adjusted | Adjusted |
| Gender |  |  |  |  |
| Female | Ref | Ref | Ref | Ref |
| Male | 0.00 (0.01) | 0.00 (0.01) | 0.00 (0.01) | 0.00 (0.01) |
| Education |  |  |  |  |
| No qualification |  |  | Ref | Ref |
| Intermediate |  |  | -0.04 (0.01)*** | -0.04 (0.01)*** |
| High |  |  | -0.05 (0.01)*** | -0.05 (0.01)*** |
| Wealth |  |  |  |  |
| Lowest quantile |  |  | Ref | Ref |
| 2n quantile |  |  | -0.03 (0.01)* | -0.03 (0.01)* |
| 3rd quantile |  |  | -0.04 (0.01)*** | -0.04 (0.01)*** |
| 4th quantile |  |  | -0.05 (0.01)*** | -0.04 (0.01)*** |
| Highest quantile |  |  | -0.04 (0.01)*** | -0.04 (0.01)*** |
| Age (continuous) | 0.00 (0.00) |  | 0.00 (0.00) |  |
| Age dummies |  | Adjusted |  | Adjusted |
| N | 5314 | 5314 | 4958 | 4958 |

Note: The binary measure of cognitive impairment or probable dementia (yes/no) was derived from modified Telephone Interview of Cognitive Status, with normal cognition coded as 0 (original score 12-27) and as 1 (original score 0-11) for cognitive impairment or probable dementia. Clustered standard errors by age and region in parentheses. * p<0.05, ** p<0.01, *** p<0.001

Supplementary Table S4. Two-Stage Least Squares estimation of second-stage regression with cognitive impairment and probable dementia (binary) as the outcome

|  | **Model 1** | **Model 2** | **Model 3** | **Model 4** |
| --- | --- | --- | --- | --- |
|  | **ß (SE)** | **ß (SE)** | **ß (SE)** | **ß (SE)** |
| Number of missing teeth | -0.08 (0.10) | -0.04 (0.09) | -0.10 (0.11) | -0.07 (0.10) |
| Region dummies | Adjusted | Adjusted | Adjusted | Adjusted |
| Gender |  |  |  |  |
| Female | Ref | Ref | Ref | Ref |
| Male | 0.00 (0.01) | 0.00 (0.01) | 0.01 (0.01) | 0.01 (0.01) |
| Education |  |  |  |  |
| No qualification |  |  | Ref | Ref |
| Intermediate |  |  | -0.07 (0.03)* | -0.06 (0.03)* |
| High |  |  | -0.09 (0.04)* | -0.08 (0.04)* |
| Wealth |  |  |  |  |
| Lowest quantile |  |  | Ref | Ref |
| 2nd quantile |  |  | -0.07 (0.04) | -0.05 (0.04) |
| 3rd quantile |  |  | -0.09 (0.05) | -0.07 (0.04) |
| 4th quantile |  |  | -0.11 (0.06) | -0.09 (0.05) |
| Highest quantile |  |  | -0.11 (0.06) | -0.09 (0.06) |
| Age (continuous) | 0.00 (0.00) |  | 0.00 (0.00) |  |
| Age dummies |  | Adjusted |  | Adjusted |
| N | 5314 | 5314 | 4958 | 4958 |
| F-statistic | 10.28 | 12.64 | 9.54 | 11.13 |

Note: The binary measure of cognitive impairment or probable dementia (yes/no) was derived from modified Telephone Interview of Cognitive Status, with normal cognition coded as 0 (original score 12-27) and as 1 (original score 0-11) for cognitive impairment or probable dementia. Clustered standard errors by age and region in parentheses. * p<0.05, ** p<0.01, *** p<0.001
